# A Method for Determining the Coefficients of Inter-Yarn Friction in Sateen Fabric with ZnO Nanowires

**DOI:** 10.3390/ma18235463

**Published:** 2025-12-04

**Authors:** Yanyan Chu, Yue Zhang, Chenhui Jiao, Baokun Zhu, Jingyu Xu, Weihan Huang, Long Gao, Xiaogang Chen

**Affiliations:** 1 Zhongyuan University of Technology, Zhengzhou 450007, China; 2 The University of Manchester, Manchester M13 9PL, UK; xiaogang.chen@manchester.ac.uk

**Keywords:** coefficients of inter-yarn friction, linear method, nanowire, sateen fabric, yarn pull-out

## Abstract

Zinc oxide nanowires are often used to improve the bulletproof performance of high-performance fabrics, but determining the coefficients of inter-yarn friction (CIFs) of those fabrics in numerical ballistic models is a challenge. In this article, the linear method is adopted to obtain the CIF of sateen fabrics with two thread densities treated with zinc oxide nanowires. For treated sateen fabrics with a thread density of 8 ends/cm (S-8-ZnO), the coefficient of static friction (CSF) and coefficient of kinetic friction (CKF) obtained by the linear method are 1.85 and 1.83, respectively. For treated sateen fabrics with a thread density of 13 ends/cm (S-13-ZnO), the CSF and CKF obtained by the linear method are 0.76 and 0.74, respectively. The obtained coefficients are input into the yarn pull-out models of the above two types of sateen fabrics. It is found that for both S-8-ZnO and S-13-ZnO fabrics, the errors of the yarn pull-out force by the linear method are 0.43% and 6.56%, respectively. The method presented in this study provides a more feasible approach for determining the CIF of chemically treated fabrics in future FE simulations.

## 1. Introduction

Aramid fabrics are widely recognized as common materials for soft bulletproof applications. Regarding fabric structure, Islam et al. [1] highlighted that satin/sateen fabrics surpass plain fabrics in performance due to their longer floating yarns, which facilitate faster stress propagation across the fabric. Further research by Chu et al. [2] compared the ballistic performance of sateen, plain, and unidirectional (UD) fabrics. Their findings revealed that sateen fabric exhibits superior ballistic performance, particularly when the number of layers and the CIF are increased. This is attributed to its greater interpenetration compared to UD fabric and the increased number of straight joints compared to plain fabric, both of which contribute to energy absorption and dispersion. In addition to its ballistic advantages, satin/sateen fabric is also superior in terms of comfort. Limeneh et al. [3] noted that satin/sateen structures offer higher air permeability, better water absorption, and lower thermal resistance and stiffness compared to twill and plain structures. These properties make satin/sateen fabrics particularly suitable for flexible body armor, meeting the dual requirements of softness and comfort. Thus, satin/sateen aramid fabrics represent an optimal choice for applications demanding both high ballistic protection and wearer comfort.

The CIF is one of the critical factors influencing ballistic performance [4]. Wang et al. [5] investigated the effect of the CIF on fabric ballistic performance using finite element (FE) simulation. Their findings indicated that within a certain range, increasing the inter-yarn friction coefficient enhanced the fabric’s bulletproof performance. Building on these results, researchers have explored various methods to increase the inter-yarn friction in fabrics. Shear thickening fluid (STF) has emerged as an effective energy-absorbing material solution due to its unique properties, while nanomaterials have gained significant attention in this field owing to their lightweight characteristics [6]. Research demonstrates that STF treatment can remarkably enhance the frictional performance of Kevlar fabrics, with yarn pull-out force increasing by 213.2% compared to untreated samples [7]. Notably, aramid fabrics treated with B_4_C-modified STF exhibit superior ballistic protection performance, showing an increase in ballistic limit velocity from 114 m/s to 179 m/s (a 57% improvement). Further structural optimization reveals that a multi-layer configuration combining one layer of STF-treated fabric with five layers of untreated fabric achieves optimal energy absorption efficiency [8]. In addition to STF treatment, other surface modification techniques have also proven effective in enhancing fabric performance. For instance, the glancing angle deposition technique has been employed to deposit aligned silver nanorods (AgNRs) onto aramid fabrics, significantly enhancing inter-yarn friction and improving ballistic performance. This nano-coating maintains the fabric’s flexibility and weight while increasing the inter-yarn friction force by 130% [9]. Similarly, LaBarre et al. [10] demonstrated that incorporating multi-walled carbon nanotubes (MWNTs) into aramid fibers increases the inter-yarn force by 230%. Their study showed that Kevlar^®^ treated with MWNTs exhibited a 15% increase in yarn modulus, a 30% increase in the CKF, a 30% increase in yarn pull-out force, and an increase of approximately 50% in ballistic limit. Shao et al. [11] further explored the friction properties of aramid woven fabrics modified with carboxylated cellulose nanofibers (CNF-C). Their experiments revealed that CNF-C modification significantly improved yarn pull-out force and fiber surface roughness. The optimal conditions—immersion for 0.5 h and a CNF-C concentration of 1 wt%—resulted in a pull-out force of 13.3 N, representing a 133.3% increase compared to untreated fabrics. These advancements highlight the potential of nanomaterials in enhancing the ballistic performance of aramid fabrics while keeping them lightweight and flexible.

Among the various nanomaterials available for surface modification, ZnO nanomaterials have garnered significant attention due to their ease of processing, low toxicity, cost-effectiveness, and compatibility with industrial-scale production. Xu et al. [12] investigated the ballistic properties of ZnO-modified aramid fabrics and found that the ballistic limit velocity of ZnO-treated two-layer and three-layer fabrics increased by 16% and 35.6%, respectively, while their surface density decreased by 45.9% and 27.9%. The growth of ZnO nanowires on aramid fibers significantly enhances inter-yarn friction, resulting in peak yarn pull-out force and energy absorption that are 10.85 times and 22.70 times greater than untreated fabrics, respectively [13]. Malakooti et al. [14] demonstrated that ZnO-coated fabrics can effectively modulate inter-yarn friction and impact resistance, with the maximum impact force of a single aramid fabric improving by approximately 66% within a specific impact velocity range.

Further advancements have been made by Majumdar et al. [15], who developed ZnO nanostructures on Kevlar^®^ fabrics, achieving an 85% increase in tensile strength for Kevlar^®^ fibers and a 24% increase for their composites. Additionally, the impact energy absorption of Kevlar^®^ fibers and their composites have been improved by 60% and 35%, respectively. Hazarika et al. [16] grafted ZnO nanorods onto woven Kevlar^®^ fabrics, enhancing the interfacial strength of Kevlar^®^/polyester resin composites and achieving up to a 60% increase in impact energy absorption. Steinke et al. [17] explored the effects of zinc oxide nanowires (ZnO NWs) grown on UHMWPE woven fabrics, reporting a 663.5% increase in friction and an 822.9% increase in pull-out energy. These modifications led to a 59.13% increase in the V_50_ ballistic limit and a 227% increase in energy absorption compared to untreated fabrics. Arora et al. [18] analyzed the impact response of ZnO nanorod-grafted UHMWPE woven fabrics, noting significant increases in inter-yarn friction (44% to 329%) and yarn pull-out force. Castellanos et al. [19] studied the low-velocity impact performance of ZnO nanowire-modified fabrics, observing strong resistance to damage during impact tests. Importantly, the flexibility of Kevlar^®^ fabric remained unchanged after ZnO nanowire coating, while the yarn pull-out force and energy under 100 N tension were increased by 266% and 293%, respectively [18]. These findings underscore the potential of ZnO nanomaterials to enhance the mechanical and ballistic properties of fabrics while maintaining their flexibility and lightweight characteristics, making them a promising candidate for advanced protective materials.

To simulate the ballistic performance of fabrics modified with nanomaterials through the FE method, we usually need to input the CIF. Chu et al. [2] used the Coulomb friction to determine the CIF after TiO_2_/ZnO treatment. Khodadadi et al. [20] and Alikarami et al. [21] developed an analytical model for calculating the CIF in STF fabric based on the yarn pull-out force, number of crossovers in the direction of the pulled yarn, and normal load at each crossover. Previous studies have employed different approaches to determine the CIF in ballistic fabrics. López-Gálvez et al. [22] proposed the linear method, which assumes a range of the CIF with specific gradients. These assumed coefficients are then input into a yarn pull-out finite element model, and linear regression is used to establish the relationship between the CIF and peak pull-out force, ultimately determining the friction coefficient range for modified plain weave fabrics. A novel measurement method was developed for carbon fiber bundles, detecting micro-forces of approximately 2 mN during fiber extraction while accounting for the bundle packing state (void fraction) and compressive stress (3–9 kPa). Stable measurements were obtained at fiber bundle volume fractions between 0.4 and 0.5, yielding a dynamic friction coefficient of 0.13 ± 0.1. Furthermore, based on Howell’s power law [23], Vu et al. [24] proposed an anisotropic friction model. By employing a constant-force spring device to maintain a consistent contact area, they achieved a precise measurement of the CIF. The experimental study utilized a combination of carbon fibers and glass fibers. Tests conducted under both dry and wet conditions revealed that both the yarn intersection angle and normal force significantly influenced the friction coefficients. Notably, under wet conditions, the capillary forces generated by water bridges between fibers resulted in a higher CIF compared to dry conditions.

It should be noted that existing studies have primarily focused on plain weave fabrics. Currently, no studies that evaluate the CIF of surface-modified aramid sateen fabrics have been found. This paper aims to accurately determine the friction coefficients for sateen fabrics, providing valuable reference data for further research in ballistic protection and related fields. The findings will contribute to a better understanding of sateen fabric behavior under ballistic impact and support the development of more accurate simulation models for protective applications.

## 2. Materials

The sateen weave fabrics are made of aramid filaments, supplied by Yantai Tayho Advanced Materials Co., Ltd. (Yantai, China). Two different warp and weft thread densities (8 and 13 ends/cm) were selected to ensure comprehensive experimental data. The required chemical reagents included anhydrous ethanol (≥99.7% purity), supplied by Tianjin Fu Yu Fine Chemical Co., Ltd. (Tianjin, China), ammonia solution (analytical grade), supplied by Xilong Scientific Co., Ltd. (Shantou, China), polyethylenimine (M.W. 600, 99%), supplied by Shanghai Macklin Biochemical Co., Ltd. (Shanghai, China), anhydrous zinc chloride (analytical grade), supplied by Shanghai Macklin Biochemical Co., Ltd. (Shanghai, China), sodium hydroxide, supplied by Tianjin Zhi Yuan Chemical Reagent Co., Ltd. (Tianjin, China), and deionized water. The detailed sample specifications are listed in Table 1. The linear density of the yarn is 840D with 2000 filaments. The fiber carrier increase percentage after growing ZnO nanowires is lower than 3%. There are four fabric specimen, S-8-U, S-8-ZnO, S-13-U, and S-13-ZnO. In the abbreviation, S stands for sateen fabric and the number means the thread density. U means the fabric is original and ZnO means the fabric has had ZnO nanowires grown on it.

## 3. Experiments and the FE Simulation Model

### 3.1. ZnO Nanowire Growth

**Figure 1:**
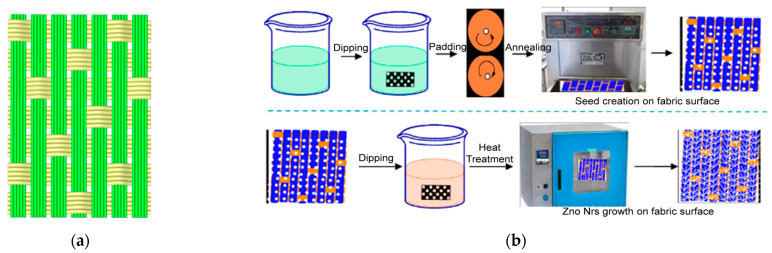
(**a**) The sateen fabric weaving structure; (**b**) the process of ZnO nanowire growth.

The growth of ZnO nanowires on the aramid sateen fabric followed a hydrothermal method, as described in Reference [25], which consists of the following two main stages: seed layer deposition and nanowire growth. The whole process is shown in Figure 1. For the seed layer deposition stage, a seed solution was prepared by dissolving sodium hydroxide (ethanol ratio of 1:2.5) and polyethylenimine (ethanol ratio 1:8) in anhydrous ethanol. Additionally, anhydrous zinc acetate was incorporated as the zinc salt precursor at a molar concentration of 0.013 mol/L. The solution was subsequently diluted and stirred at 65 °C. The sateen fabric was immersed in the seed solution for 20 min, then subjected to a dip-pad-dry-cure process (two dips and two pads) and baked at 150 °C for 18 min. For the nanowire growth stage, a mixture of anhydrous zinc acetate, ammonia solution, polyethylenimine, and deionized water was reacted at 86 °C for 2 h, followed by air drying. The surface morphology of the aramid fabric was characterized using a ZEISS high-resolution field-emission scanning electron microscope (FESEM) (ZEISS, Oberkochen, Germany). All tests were conducted at a controlled room temperature of 22 ± 2 °C.

### 3.2. The Inter-Yarn Friction Test

**Figure 2:**
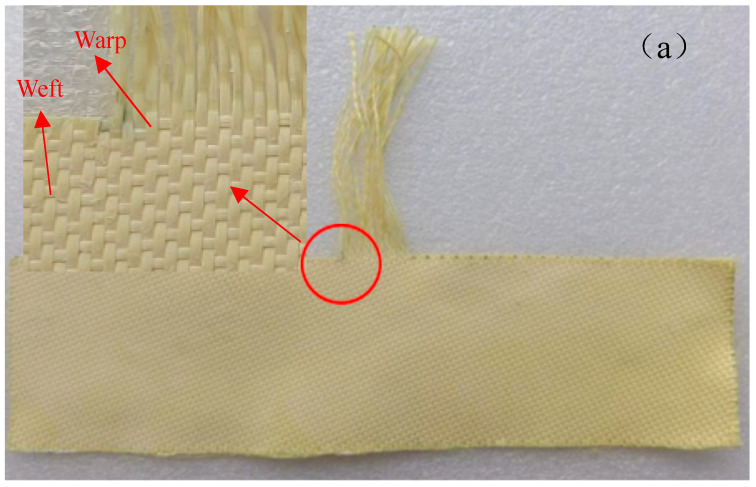

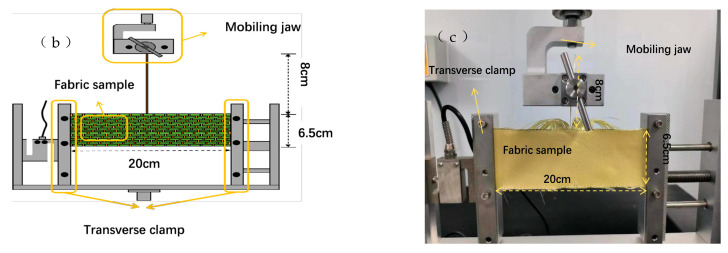
Yarn pull-out test: (**a**) identification diagram of warp and weft yarns, (**b**) schematic diagram, and (**c**) testing image.

The inter-yarn friction property is commonly characterized by the yarn pull-out force [9]. The test for obtaining the yarn pull-out force is called the yarn pull-out test and it was conducted on an AI-7000S1 test machine (Gotech Testing Machines Inc., Taichung, Taiwan, China) with a range of 20 N. Figure 2 shows the test device and the clamp of the sample. This experiment was performed with reference to the literature [8,22,26]. The sample has an overall “convex” shape, with a length of 20 cm and a width of 14.5 cm. The arrangements of the warp and weft yarns are identified in Figure 2a. As shown in Figure 2b,c, there is a section of exposed yarn, 8 cm long, at the top of the sample. During the testing process, the two sides of the sample need to be firmly clamped, and at the same time we need to ensure that the transverse tension is 100 newtons. Next, we select the yarn located at the center of the sample, use the upper clamp to grip its top end, and carefully control the clamping distance to ensure it is 1 cm. After completing the above operations, testing can commence. For sateen fabrics, pull-out tests should be conducted separately both before and after the growth of nanowires. During the testing process, the upper clamp will move at a speed of 500 mm per minute. Moreover, each type of sample should be tested 10 times repeatedly. In addition, the temperature of the samples was 27.6 °C and the humidity was 25.1% both before and after the experiment.

### 3.3. Numerical Simulation for the Yarn Pull-Out Test

#### 3.3.1. Model Building

**Figure 3:**
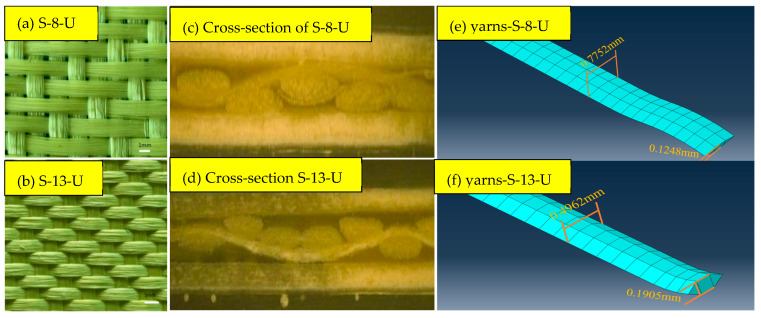
The structure of aramid sateen fabric. (**a**) Actual S-8-U; (**b**) actual S-13-U; (**c**) cross-section of S-8-U; (**d**) cross-section of S-13-U; (**e**) mesh yarns-S-8-U; and (**f**) mesh yarns-S-13-U.

Using Abaqus software (version 2016), a yarn pull-out simulation model is built. This model is designed to predict the inter-yarn friction of different sateen aramid fabrics, which are used to compare with the above yarn pull-out test results. The core components include the fabric sample, the moving jaw, and the transverse clamp. Figure 3 shows the fabric and yarn geometry. Figure 3a,b shows the fabric structure of S-8-U and S-13-U, respectively, which are 5-end fabrics with a step of 3. Figure 3c,d shows a cross-section view of the fabric observed by ultra-depth-of-field microscopy. The cross-section of one yarn almost takes the shape of a convex lens. The widths of the cross-sections are 0.7752 mm and 0.4962 mm, respectively. The fabric is modeled at the yarn geometry level and is generated using the sweep method. Those yarns are assembled into a single layer of fabric with a size of 20 cm × 6.5 cm according to the actual sateen weave structure. Table 2 shows the parameters used to generate the “yarn pull-out model”. The pulling yarn with a length of 14.5 cm is placed in the center. This is consistent with actual yarn pull-out tests. In the simulation, the fabric edge is fixed by the transverse clamp based on the actual size, and the constraint is realized by binding. In addition, the moving jaw is simplified into a small gray rigid block, the shape of which matches the cross-section of the yarn. It is regarded as an undeformable body. The end of the pulling yarn and the small rigid block are connected by binding and bonding to realize synchronous movement. The rigid block is given a specific speed and moves at a rate of 500 mm/min in the positive direction along the Z-axis, causing the pulling yarn to move synchronously.

**Figure 4:**
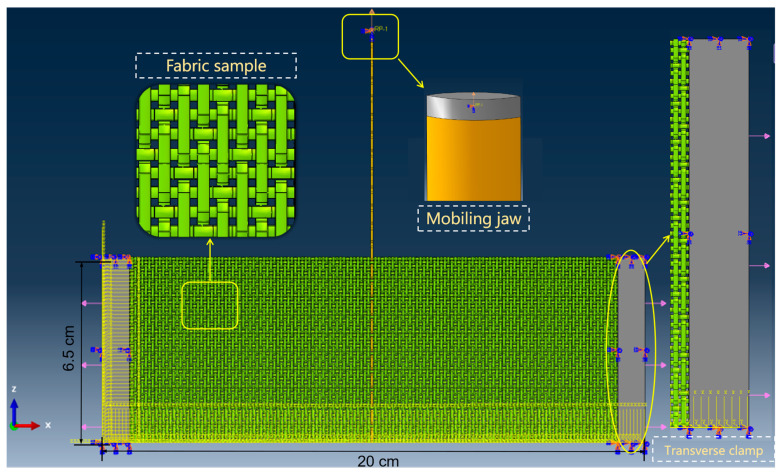
Yarn pull-out model of sateen fabric.

The mesh volume for the yarn, transverse clamp, and moving jaw is set to 0.00023 m^3^, 0.00023 m^3^, and 0.00016 m^3^, respectively. In Abaqus simulation, the yarn is modeled as a continuum with a solid cross-section, which has high bending stiffness and inevitably leads to the overestimation of the yarn pull-out force. Besides, aramid yarn is a collection of hundreds of loose and soft filaments, without twisting or twisting between the filaments. During the pulling process, they can slide relative to each other so that the yarn does not undergo significant stress changes through bending deformation. To address this issue, the yarn is idealized using hexahedral elements (C2D6R) with two elements through the thickness, as shown in Figure 3e,f. This is a common method when considering bending resistance or bending stiffness using solid elements with reduced integration. For the meshing mode of the transverse clamp and the rigid block of the moving jaw, C3D8R elements are used. The whole yarn pull-out model is shown in Figure 4.

#### 3.3.2. The Mechanical Properties

**Figure 5:**
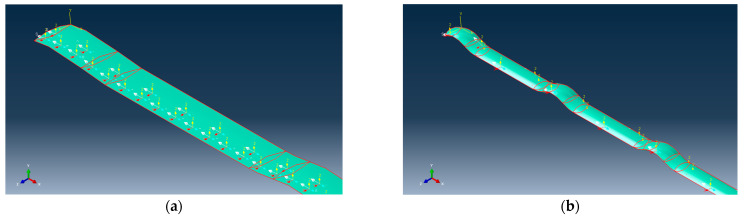
The designated direction of the yarn’s mechanical properties. (**a**) S-8-U; (**b**) S-13-U.

Table 3 shows the specific parameters of the material properties of aramid yarns. The aramid yarn is regarded as an orthogonal anisotropy material. The E1 is obtained through yarn tensile experiments, assuming that the X-direction is along the fiber axis. The transverse elastic moduli (E2 and E3) and shear moduli (G12, G13, and G23) are approximately one order of magnitude smaller than E1. The elastic properties of these yarn materials must be strictly defined in the material direction, as shown in Figure 5. For Poisson’s ratio, set Nu12 = Nu13 = Nu23 = 0, given the insignificant impact of lateral deformation on the yarn’s mechanical response. The direction of 1, 2 and 3 are shown in Figure 5. As the yarn is a loose collection of individual filaments, its thread density is determined by the content in the yarn^®^. The fiber packing density was set to 1248 kg/m^3^, with a packing factor of 0.86 applied to account for the voids within the yarn structure. The transverse tension fixture plate and the moving fixture are both made of steel material, with a mass density of 7687 kg/m^3^, a Young’s modulus of 210 GPa, and a Poisson’s ratio of 0.3.

## 4. Results

### 4.1. SEM Morphology Characterization

**Figure 6:**
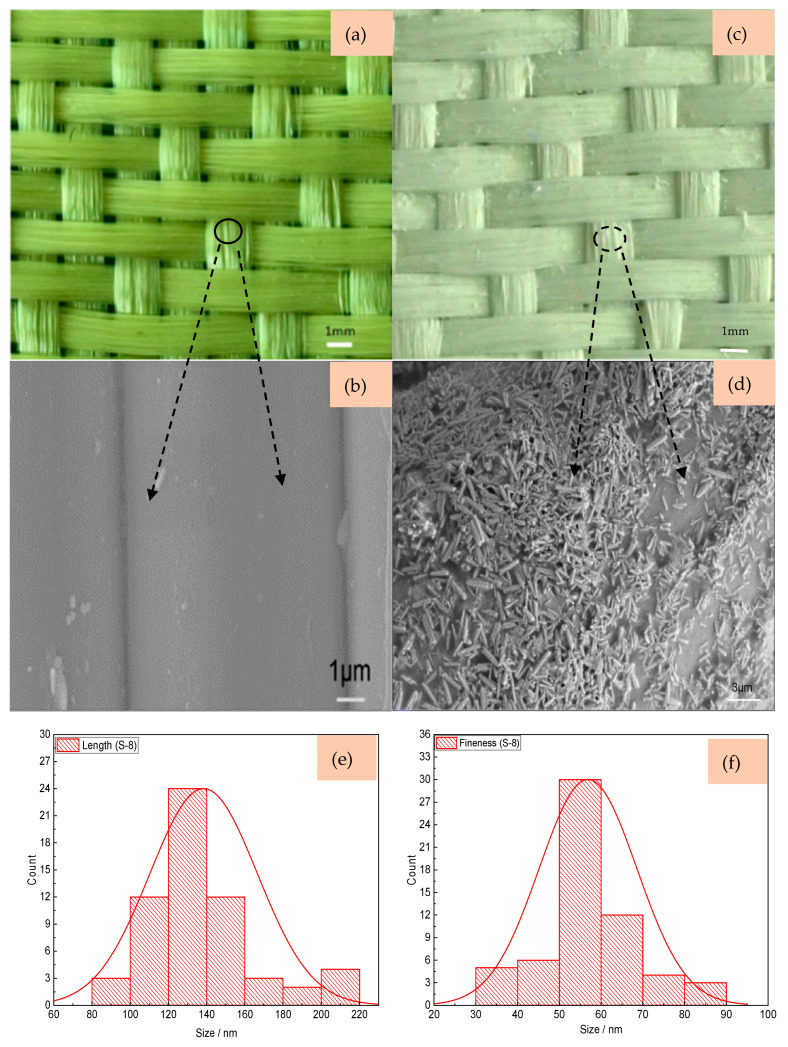
The morphology of S-8 fabric. (**a**) S-8-U; (**b**) SEM image of S-8-U; (**c**) S-8-ZnO; (**d**) SEM image of S-8-ZnO; (**e**) the length of ZnO nanowires on S-8-ZnO; and (**f**) the fineness of ZnO nanowires on S-8-ZnO.

**Figure 7:**
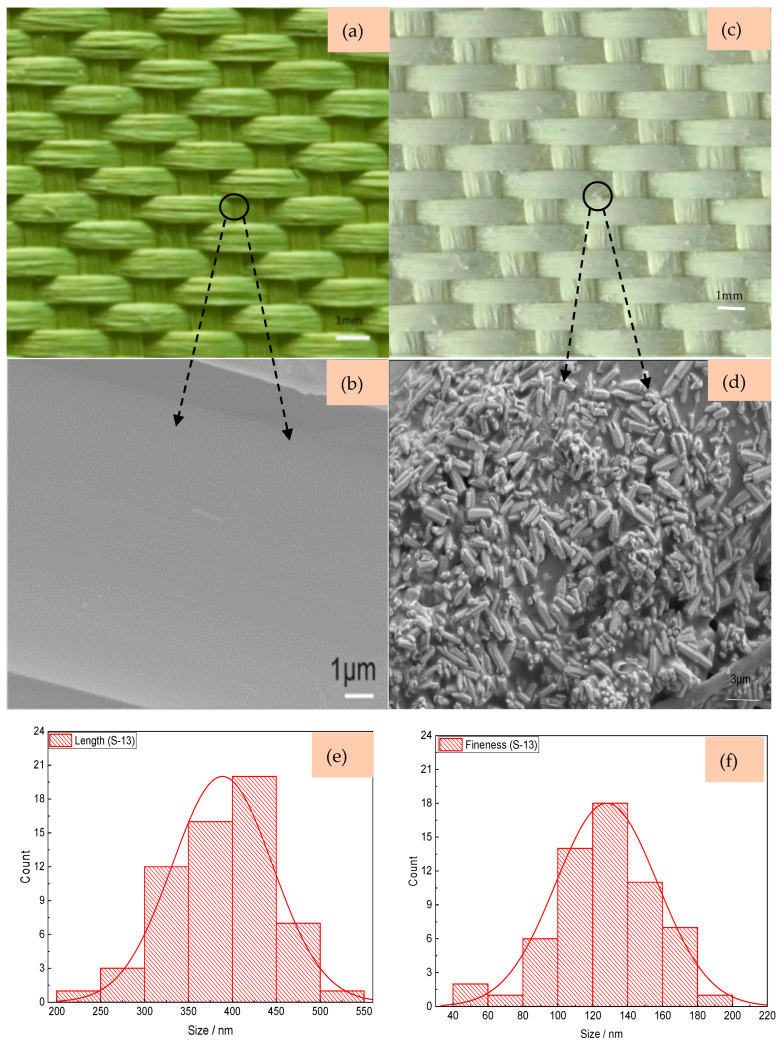
The morphology of S-13 fabric. (**a**) S-13-U; (**b**) SEM image of S-13-U; (**c**) S-13-ZnO; (**d**) SEM image of S-13-ZnO; (**e**) the length of ZnO nanowires on S-13-ZnO; and (**f**) the fineness of ZnO nanowires on S-13-ZnO.

Figure 6 shows the morphology of filaments in two sateen fabrics with and without ZnO nanowires grown. In the S-8-U fabric, from the images in Figure 6a, there are some gaps between the fiber filaments. It is found that the surfaces of the filaments in the S-8-U aramid sateen fabric are relatively smooth, forming a “rod-like” shape. After nanowire grow, the overall color of S-8-ZnO is a uniform “silver white”. Compared with the S-8-U fabric, the surfaces of the filaments in S-8-ZnO are covered with more nanowires. Based on the analyses in Figure 6e,f, the nanowires of the S-8-ZnO fabric are thinner, where the averaged length of the nanowires on S-8 fabric is 138.41 nm and the average fineness is 56.61 nm. Due to the larger thread density of the S-13-U fabric, its structure is tighter compared with S-8-U fabric, as shown in Figure 7a. Figure 7b shows that the surface of S-13-U fabric is also quite smooth. From Figure 7c,d, it can be observed that the surface of S-13-ZnO is covered with ZnO nanowires that adhere to each other, filling the gaps between the aramid filaments. Compared with the nanowires on S-8-ZnO, the nanowires on S-13-ZnO fabric are slightly thicker. Figure 7e,f shows that the averaged length and fineness of nanowires on S-13 fabric are 388.41 nm and 128.02 nm, respectively.

### 4.2. The Validation of the Yarn Pull-Out Model

**Figure 8:**
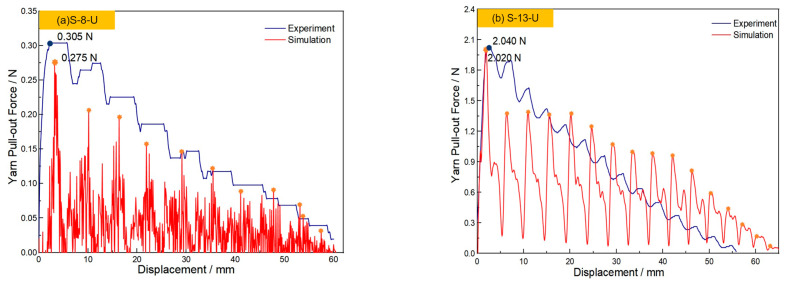
The experimental and simulation results of yarn pull-out force. (**a**) “S-8-U” fabric; (**b**) “S-13-U” fabric.

Table 4 shows the yarn pull-out force test results of four fabric samples under a transverse tension of 100 N. Figure 8 shows the test and simulated yarn pull-out curves of force- displacement in two original fabrics under 100 N transverse tension, respectively. The CIF is represented by the coefficients of static (μ_s_) and kinetic friction (μ_k_). Based on previous investigations, the values of μ_s_ and μ_k_ for the original aramid filament are set to 0.18 and 0.16, respectively. The CIFs for the two original sateen fabrics are the same since the yarns are same. The numerical simulation peak values for S-8-U and S-13-U were 0.275 N and 2.020 N, respectively, showing deviations of 9.836% and 0.980% compared to the experimental measurements of 0.305 N and 2.040 N. Notably, both error margins were maintained within the 10% threshold, demonstrating satisfactory agreement between simulation and experimental data. At the same time, in the two original fabrics, the oscillation trend of the pull-out force as a function of the displacement also tends to be similar. As the pulled yarn moves forward, the pull-out force-to-displacement curve will fluctuate accordingly, and the pull-out force will gradually decrease. The oscillation frequency is also consistent with the experiment, indicating that the finite element analysis of the yarn pull-out model for original sateen fabric shows good consistency with the experimental results.

**Figure 9:**
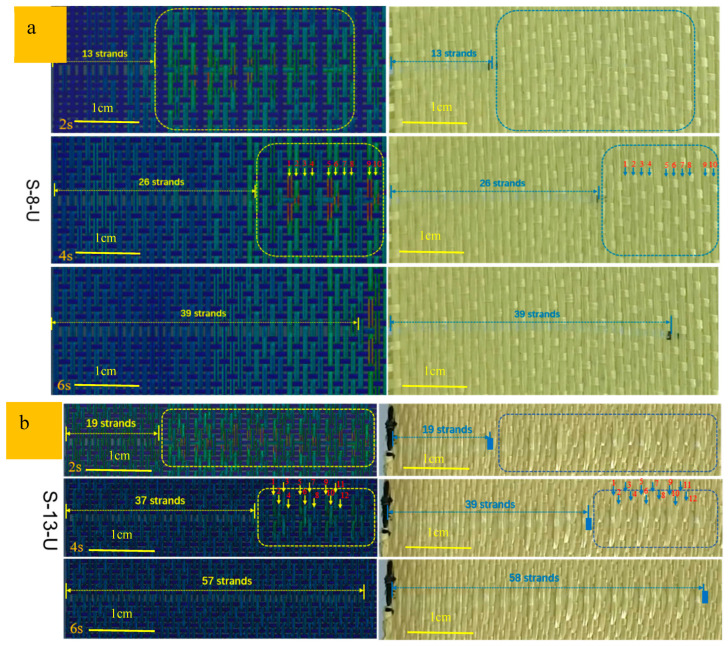
Comparison of the pull-out process between simulation and experiment. (**a**) “S-8-U” fabric; (**b**) “S-13-U” fabric.

Figure 9 shows the specific displacement of pulled-out yarns for S-8-U and S-13-U fabrics in simulation and experiment, respectively. From 2 s to 6 s, the pulled-out yarn keeps moving, and the amount of displacement that the yarn is drawn out correspondingly increases. At the same time, the yarns around the pulled-out yarn experience some stress, and the cloud map distribution in the simulation is consistent with the actual distribution. In the S-8-U fabric, every 2 s, the number of yarns pulled out in the simulation and the experiment was exactly the same, as follows: 13, 26, and 39, respectively. In 4 s, the stress in the pull-out yarn area is lower, while the stress in the unpulled area is higher, and the stress in some yarns is particularly significant. In practical experiments, this phenomenon corresponds to the protrusion and constraint of yarn fiber bundles. In the S-13-U fabric, these phenomena are more obvious, but the number of yarns extracted in the simulation and the experiment is slightly different, with about 1–2 more yarns extracted in the experiment. This may be due to the tight assembly of the yarn in the simulation, while the actual yarn can be squeezed and aggregated during weaving and pulling out. Nevertheless, the simulation and experimental results still show consistency and validity, which provides strong support for the study of the inter-yarn friction property of sateen fabric.

## 5. Discussion

### 5.1. The Range of the CIF of Fabric with ZnO Nanowire

The linear method was implemented with reference to the methodology established by López-Gálvez et al. [22]. The difference is that they did not distinguish whether the CIF is the CSF or the CKF, but the current study employs two pairs of CIFs. In addition, their fabric is plain fabric while the present one has a sateen structure. The method involves establishing a combination of the CSF and CKF for Sateen-8-ZnO fabric with a certain gradient based on the original CSF and CKF for S-8-U, and similarly establishing another combination of the CSF and CKF for S-13-ZnO fabric. Since the original filament yarns in the two fabrics are same, both the CSF and the CKF for the two original fabrics are set to 0.18 and 0.16. Based on this CSF and CKF, two groups of assumed CSFs and CKFs are set for the two sateen fabrics with ZnO nanowires grown on them, as shown in Table 5. These data are input into the established yarn pull-out model, and through the simulation process, the inter-yarn friction curves are obtained.

**Figure 10:**
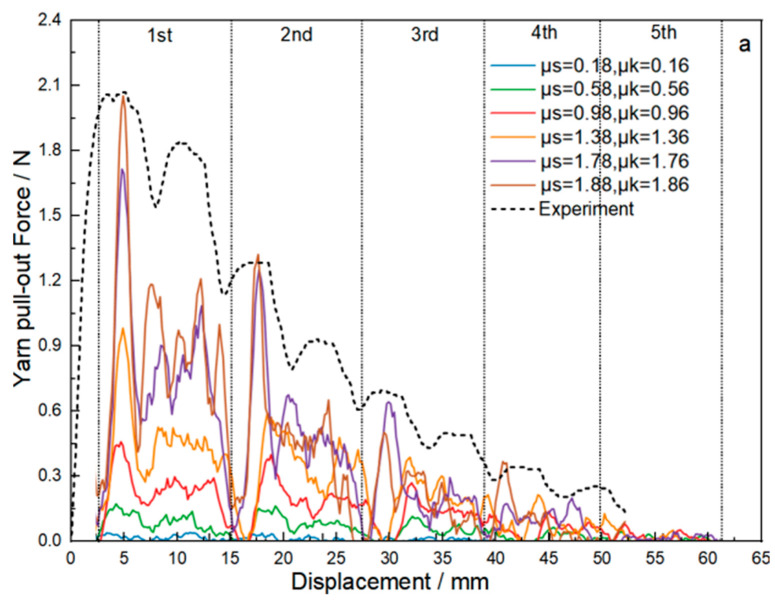

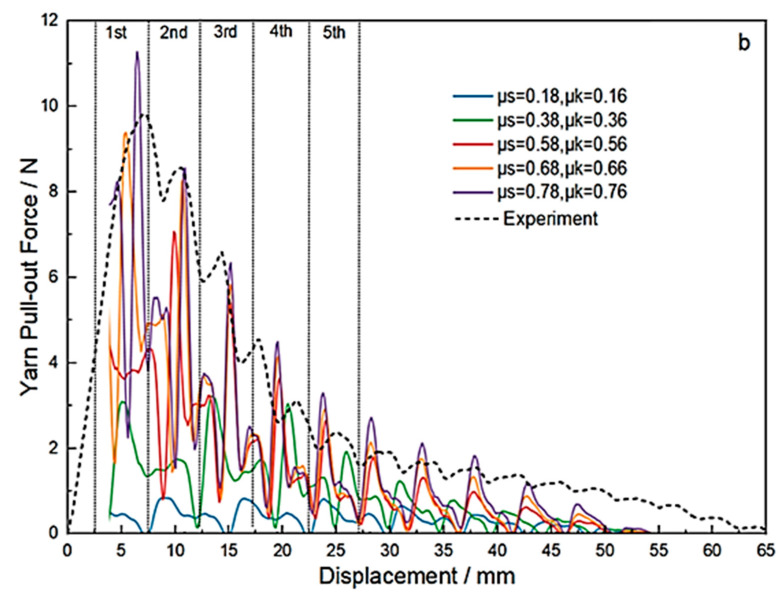
The simulated yarn pull-out force to displacement under the assumed CIF. (**a**) “S-8-ZnO” model; (**b**) “S-13-ZnO” model.

Figure 10 shows the yarn pull-out curve of force to displacement under different assumed CSFs and CKFs for the two thread density sateen fabrics. As the CIF increases, the yarn pull-out force also increases. The explanation is that the higher CIF means a rougher surface and thus it would obstruct the yarn to be pulled out. In addition, the pull-out force decreases in steps as displacement increases. This is because as the length of the yarn being pulled out becomes longer, fewer yarns will be drawn out and interwoven with it, so the force will decrease. Comparing the yarn pull-out force curves of S-8 fabric to those of S-13-U fabric, the trends of the curves are slightly different, as the curves of S-13-U fabric are steeper, with more obvious peaks, and the pull-out force is greater than that of S-8-U fabric. The main reason is that once the pulled yarn passes through a crossing point, oscillation occurs and the curve produces peaks. Since S-13-U fabric has a larger number of warp and weft crossing points and a higher degree of bending, its curves present more peaks and higher peak values. In addition, a comparative analysis of ZnO-treated fabrics revealed that the CSF/CKF ranges for S-13-ZnO and S-8-ZnO were 1.78–1.88/1.76–1.86 and 0.68–0.78/0.66–0.76, respectively, demonstrating a significant density-dependent sensitivity for the CIF, which is attributed to the tighter structure obstructing yarn movement.

### 5.2. The Specific CIF Value of Fabric with ZnO Nanowire

**Figure 11:**
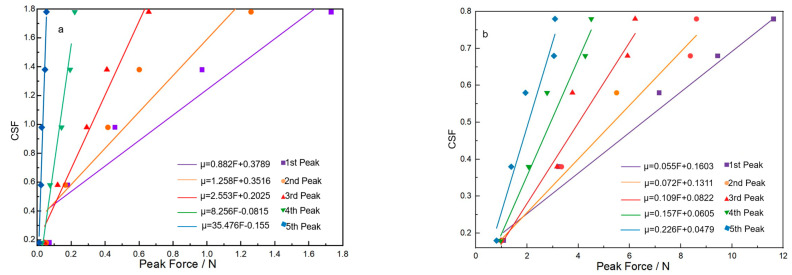
Linear trend line of peak value to CSF for the two fabrics. (**a**) S-8-ZnO fabric model; (**b**) S-13-ZnO fabric model.

**Figure 12:**
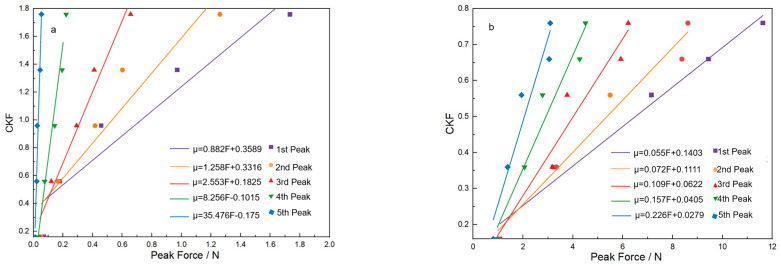
Linear trend line of peak value to CKF for the two fabrics. (**a**) S-8-ZnO fabric model; (**b**) S-13-ZnO fabric model.

Although the range of the CIF of the sateen fabric with ZnO nanowire growth is roughly determined, its specific value is still not confirmed. Therefore, the linear regression method is adopted. Table 6 shows that the five peak force values at different CIFs for the two fabrics are picked out from Figure 10. The corresponding linear relationship between the CIF and peak force is shown in Figure 11 and Figure 12, respectively. Figure 11 and Figure 12 show the relationship of the CSF and CKF to the peak value for the two fabrics with ZnO, respectively. Through the linear regression between the CIF data and the different peak force values, the linear regression equation and the linear line are obtained for each peak value, as shown in Figure 11 and Figure 12. For the linear regression equations constructed for each of the aforementioned peak values, statistical analysis was conducted, and the resulting statistical parameters are listed in Table 7. It is worth noting that all R^2^ values are above 0.9, indicating good fit between the CIFs and peak force values. The methodology was implemented based on the approach proposed in Reference [22]. By substituting the first to fifth peak force values at the maximum CSF for the S-8-ZnO and S-13-ZnO fabrics into the corresponding linear equations for the CSF, five μ_s_ values at corresponding peak values are obtained. Through averaging the five μ_s_ values, the final μ_s_ is determined, as shown in Table 8. In the same way, five μ_k_ values at the corresponding peak force values are also calculated. Table 9 displays the five μ_k_ values calculated and their means. By calculating the means of those μ_s_ and μ_k_ values, the CSF and CKF for the S-8-ZnO fabric are 1.85 and 1.83, respectively. The CSF and CKF for the S-13-ZnO fabric are 0.76 and 0.74, respectively.

### 5.3. The CIF Comparison of Fabrics with ZnO Nanowires Between the FE Simulation and Test

**Figure 13:**
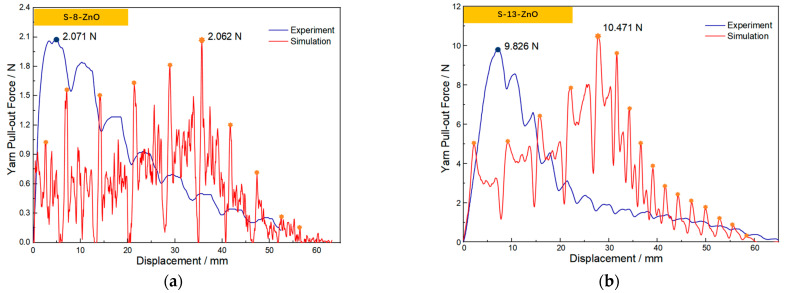
The experimental and simulation results for the yarn pull-out force by the linear method. (**a**) “S-8-ZnO” fabric; (**b**) “S-13-ZnO” fabric.

Figure 13a,b presents the experimental and simulated yarn pull-out force results using the coefficients obtained from the linear method. The differences in peak pull-out force between the simulation and experiment are only 0.43% for S-8-ZnO and 6.56% for S-13-ZnO, demonstrating that the linear method provides a significantly more accurate CIF for simulation. It is noteworthy that, compared with the experimental curve, the peak in the simulated curve appears slightly later. This discrepancy arises because the aramid yarn actually consists of thousands of fibril filaments with a loose structure, resulting in poor overall integrity. In contrast, the simulation models the yarn as a solid body with higher rigidity than the actual yarn. This increased stiffness creates greater resistance to yarn movement, delaying the peak pull-out force in the simulation. These findings confirm that the linear method is more reliable for determining the CIF in fabrics with ZnO nanowire, providing critical inputs for accurate ballistic impact simulations.

**Figure 14:**
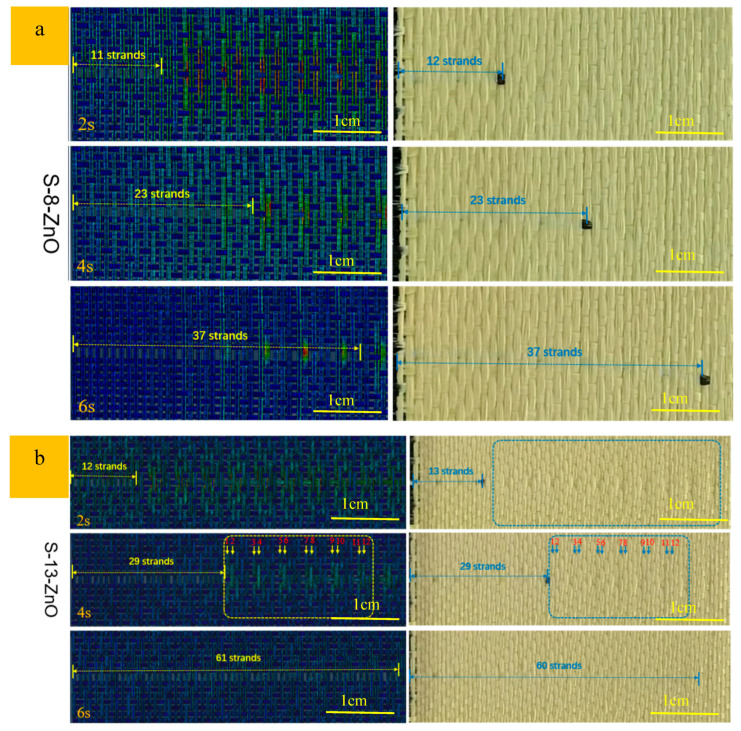
Comparison of the pull-out process between simulation by the linear method and experiment. (**a**) “S-8-ZnO” fabric; (**b**) “S-13-ZnO” fabric.

**Figure 15:**
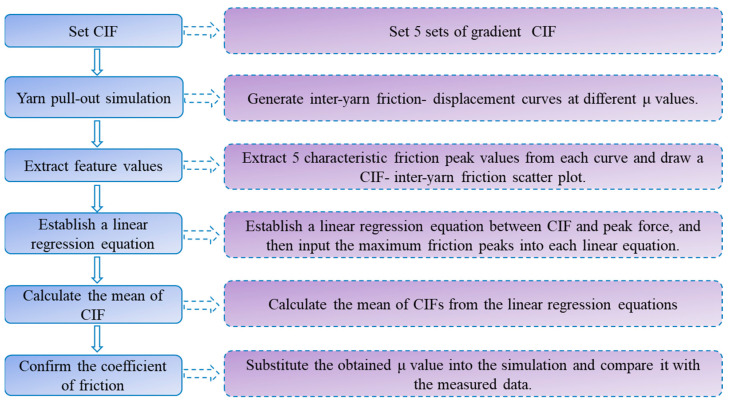
The process of determining the CIF by linear regression methods.

Figure 14 presents a comparative analysis of the yarn pull-out process between numerical simulations using linear regression-derived friction coefficients and experimental measurements for ZnO nanowire-modified sateen fabrics. Quantitative evaluation demonstrates excellent agreement between simulated and experimental results for both S-8-ZnO and S-13-ZnO fabrics as follows: the number of the yarn moving at 2 s, 4 s, and 6 s is 11, 23, and 37 for S-8-ZnO versus experimental values of 12, 23, and 37; meanwhile, S-13-ZnO shows simulated results of 12, 29, and 61 yarns compared to experimental counts of 13, 29, and 60. The maximum deviation of only one yarn validates the reliability of the CIF obtained through linear regression analysis. Experimental observations reveal that ZnO nanowire incorporation significantly enhanced inter-yarn friction, resulting in reduced cooperative yarn motion during the initial pull-out stages (e.g., a one-yarn difference for S-8-ZnO at 2 s). Progressive pull-out demonstrated that final cumulative yarn counts matches the experimental data with <1.7% error. This dynamic behavior indicates that an increase in frictional forces promotes stress localization, consequently decreasing the number of peripheral yarns participating in load transfer. The remarkable consistency between finite element simulations and experimental data (R^2^ > 0.98) conclusively verifies the accuracy of the linear regression method for the interfacial friction parameter characterization in these engineered textile systems. The whole process for determining the CIF is shown in Figure 15.

## 6. Conclusions

This paper studies the method for determining the CIF of sateen fabrics with different thread densities after ZnO nanowire growth. Through an analysis of the accuracy of the linear method, the following conclusions are drawn:

The CSF and CKF of S-8-ZnO sateen fabric obtained by the linear method are 1.85 and 1.83, respectively, while those of S-13-ZnO sateen fabric are 0.76 and 0.74, respectively. When inputting these CIFs into the yarn pull-out models of the two sateen fabrics, the error between the simulated peak pull-out force and the experimentally measured pull-out force is 0.43% for S-8-ZnO fabric and 6.56% for S-13-ZnO fabric. In summary, the linear method is suitable for evaluating the CIF of sateen fabrics treated with nanowires and is of great significance for subsequent finite element analyses of the ballistic impact. The current research has verified how to accurately obtain the CIF of sateen fabric with ZnO nanowire, which provides a key parameter input method for the finite element method to study the ballistic impact performance of modified fabric. The linear regression method established in this study is not only suitable for sateen fabric with ZnO nanowires, but it can also be applied to other fabric structures, such as plain weave, twill, and so on. In future studies, the method used in this paper can be integrated into the multi-scale finite element model, providing more accurate parameter support for ballistic impact simulations of bulletproof fabrics.

## Figures and Tables

**Table 1:** The sample prepared.

No.	Fabric Type	Areal Density (kg/m^2^)	Thread Density (ends/cm)	Fiber	Fiber Denier	Average Weight (g)	Weight Increase Percentage (%)
1	S-8-U	0.150	8 × 8	Aramid	840D	6.33	-
2	S-8-ZnO	0.160	8 × 8	Aramid	840D	6.45	1.896
3	S-13-U	0.257	13 × 13	Aramid	840D	9.97	-
4	S-13-ZnO	0.258	13 × 13	Aramid	840D	10.27	3.009

**Table 2:** Fabric parameter settings.

Fabric Sample	Thread Density (ends/cm)	Cycle Length (mm)	Fabric Thickness H (mm)	Cross-Section Width (mm)
S-8-U	8 × 8	6.410	0.1248	0.7752
S-13-U	13 × 13	3.846	0.1905	0.4962

**Table 3:** Specific parameters of yarn properties.

Material Properties	Direction 1	Direction 2	Direction 3
Elastic modulus	E1 = 101 GPa	E2 = 21 GPa	E3 = 21 GPa
Shear modulus	G12 = 20 GPa	G13 = 20 GPa	G23 = 20 GPa
Poisson’s ratio	0	0	0

**Table 4:** Yarn pull-out force test results for different fabric samples.

Sample	S-8-U	S-13-U	S-8-ZnO	S-13-ZnO
1	0.284	2.060	1.922	9.292
2	0.294	2.079	2.265	9.885
3	0.304	1.991	2.069	9.677
4	0.304	2.118	2.471	9.581
5	0.314	1.962	1.844	9.836
6	0.324	2.010	1.922	9.709
7	0.286	2.099	2.167	10.081
8	0.286	2.079	2.001	9.738
9	0.299	2.001	1.991	10.385
10	0.303	2.001	2.059	10.071
Mean	0.305	2.040	2.071	9.826
Sd	0.016	0.053	0.187	0.304
CV (%)	5.20	2.60	9.03	3.09
SE	0.0051	0.0168	0.0591	0.0961
95% CI	[0.293, 0.317]	[2.002, 2.078]	[1.937, 2.205]	[9.608, 10.044]

**Table 5:** Coefficient of friction setting of sateen fabric by the linear method.

Fabric Model	$\mu_{s}$	$\mu_{k}$
S-8-ZnO	0.18	0.16
	0.58	0.56
	0.98	0.96
	1.38	1.36
	1.78	1.76
	1.88	1.86
S-13-ZnO	0.18	0.16
	0.38	0.36
	0.58	0.56
	0.68	0.66
	0.78	0.76

**Table 6:** Five peak values in different CIF cases for two sateen fabrics with ZnO.

	Friction Coefficient	1st Peak (N)	2nd Peak (N)	3rd Peak (N)	4th Peak (N)	5th Peak (N)
S-8-ZnO	$\mu_{s}$ = 0.18, $\mu_{k}$ = 0.16	0.06892	0.05330	0.04332	0.02920	0.01128
	$\mu_{s}$ = 0.58, $\mu_{k}$ = 0.56	0.17833	0.16616	0.12042	0.07576	0.02252
	$\mu_{s}$ = 0.98, $\mu_{k}$ = 0.96	0.45653	0.41651	0.29150	0.14316	0.02633
	$\mu_{s}$ = 1.38, $\mu_{k}$ = 1.36	0.97078	0.60152	0.41053	0.19360	0.04566
	$\mu_{s}$ = 1.78, $\mu_{k}$ = 1.76	1.73333	1.25992	0.65699	0.22110	0.05411
S-13-ZnO	$\mu_{s}$ = 0.18, $\mu_{k}$ = 0.16	1.06999	0.99572	0.98008	0.96492	0.80709
	$\mu_{s}$ = 0.38, $\mu_{k}$ = 0.36	3.22062	3.34414	3.17114	2.06028	1.37907
	$\mu_{s}$ = 0.58, $\mu_{k}$ = 0.56	7.14995	5.49451	3.76871	2.78125	1.93676
	$\mu_{s}$ = 0.68, $\mu_{k}$ = 0.66	9.44210	8.37237	5.92163	4.27110	3.04738
	$\mu_{s}$ = 0.78, $\mu_{k}$ = 0.76	11.3260	8.61174	6.22010	4.50542	3.09037

**Table 7:** Linear regression statistical parameters of the fabrics’ dynamic and static friction coefficients (μ_s_ and μ_k_) under different peak values.

Sample	Peak No.	Linear Equations	R^2^	Slope CI	Intercept CI	Max F-Predicted Interval
S-8-ZnO-μ_s_	1st	μ = 0.882F + 0.3789	0.91	[0.369, 1.395]	[−0.091, 0.849]	[0.967, 2.847]
	2nd	μ = 1.258F + 0.3516	0.90	[0.542, 1.974]	[−0.116, 0.819]	[1.380, 2.494]
	3rd	μ = 2.553F + 0.2025	0.97	[2.125, 2.981]	[0.137, 0.268]	[1.717, 2.043]
	4th	μ = 8.256F − 0.0815	0.95	[6.874, 9.638]	[−0.172, 0.009]	[1.573, 1.915]
	5th	μ = 35.476F − 0.1550	0.97	[29.536, 41.416]	[−0.242, −0.068]	[1.594, 1.936]
S-8-ZnO-μ_k_	1st	μ = 0.882F + 0.3589	0.91	[0.369, 1.395]	[−0.111, 0.829]	[0.947, 2.827]
	2nd	μ = 1.258F + 0.3316	0.90	[0.542, 1.974]	[−0.136, 0.799]	[1.360, 2.474]
	3rd	μ = 2.553F + 0.1825	0.97	[2.125, 2.981]	[0.117, 0.248]	[1.697, 2.023]
	4th	μ = 8.256F − 0.1015	0.95	[6.874, 9.638]	[−0.192, −0.011]	[1.553, 1.895]
	5th	μ = 35.476F − 0.1750	0.97	[29.536, 41.416]	[−0.262, −0.088]	[1.574, 1.916]
S-13-ZnO-μ_s_	1st	μ = 0.055F + 0.1603	0.98	[0.04127, 0.06873]	[0.0576, 0.2630]	[0.63863, 0.92783]
	2nd	μ = 0.072F + 0.1311	0.97	[0.05234, 0.09166]	[0.0234, 0.2388]	[0.69012, 0.81188]
	3rd	μ = 0.109F + 0.0822	0.95	[0.08215, 0.13585]	[0.0456, 0.1188]	[0.69942, 0.82058]
	4th	μ = 0.157F + 0.0605	0.95	[0.11845, 0.19555]	[0.0289, 0.0921]	[0.70752, 0.82848]
	5th	μ = 0.226F + 0.0479	0.97	[0.17018, 0.28182]	[0.0156, 0.0802]	[0.68564, 0.80636]
S-13-ZnO-μ_k_	1st	μ = 0.055F + 0.1403	0.98	[0.04127, 0.06873]	[0.0376, 0.2430]	[0.61863, 0.90783]
	2nd	μ = 0.072F + 0.1111	0.97	[0.05234, 0.09166]	[0.0034, 0.2188]	[0.67012, 0.79188]
	3rd	μ = 0.109F + 0.0622	0.95	[0.08215, 0.13585]	[0.0256, 0.0988]	[0.67942, 0.80058]
	4th	μ = 0.157F + 0.0405	0.95	[0.11845, 0.19555]	[0.0089, 0.0721]	[0.68752, 0.80848]
	5th	μ = 0.226F + 0.0279	0.97	[0.17018, 0.28182]	[−0.0044, 0.0602]	[0.66564, 0.78636]

**Table 8:** The linear equation and the **μ_s_** calculated by these equations.

	Peak No.	Linear Equations	R^2^	Maximum Friction Force (N)	μ_s_	Mean
S-8-ZnO	1st peak	μ = 0.882F + 0.3789	0.91	1.73333	1.908	1.85
	2nd peak	μ = 1.258F + 0.3516	0.90	1.25992	1.937	
	3rd peak	μ = 2.553F + 0.2025	0.97	0.65699	1.880	
	4th peak	μ = 8.256F − 0.0815	0.95	0.22110	1.744	
	5th peak	μ = 35.476F − 0.1550	0.97	0.05411	1.765	
S-13-ZnO	1st peak	μ = 0.055F + 0.1603	0.98	11.32600	0.783	0.76
	2nd peak	μ = 0.072F + 0.1311	0.97	8.61174	0.751	
	3rd peak	μ = 0.109F + 0.0822	0.95	6.22010	0.760	
	4th peak	μ = 0.157F + 0.0605	0.95	4.50542	0.768	
	5th peak	μ = 0.226F + 0.0479	0.97	3.09037	0.746	

**Table 9:** The linear equation and the **μ_k_** calculated by these equations.

	Peak No.	Linear Equations	R^2^	Maximum Friction Force (N)	μ_k_	Mean
S-8-ZnO	1st peak	μ = 0.882F + 0.3589	0.91	1.73333	1.888	1.83
	2nd peak	μ = 1.258F + 0.3316	0.90	1.25992	1.917	
	3rd peak	μ = 2.553F + 0.1825	0.97	0.65699	1.860	
	4th peak	μ = 8.256F − 0.1015	0.95	0.22110	1.724	
	5th peak	μ = 35.476F − 0.1750	0.97	0.05411	1.745	
S-13-ZnO	1st peak	μ = 0.055F + 0.1403	0.98	11.32600	0.763	0.74
	2nd peak	μ = 0.072F + 0.1111	0.97	8.61174	0.731	
	3rd peak	μ = 0.109F + 0.0622	0.95	6.22010	0.740	
	4th peak	μ = 0.157F + 0.0405	0.95	4.50542	0.748	
	5th peak	μ = 0.226F + 0.0279	0.97	3.09037	0.726	

## Data Availability

The original contributions presented in this study are included in the article. Further inquiries can be directed to the corresponding author.
